# The “two-hit” storm: a hyper-inflammatory endotype in pediatric long COVID and its role in the severity of secondary bacterial pneumonia—a mechanistic review and clinical implications

**DOI:** 10.3389/fpubh.2026.1782871

**Published:** 2026-04-20

**Authors:** Hong Huang, Siyi Chen, Zheng Fang, Yingtao Ma, Yan Xu, Guojuan Dong

**Affiliations:** 1 Changchun University of Chinese Medicine, Changchun, Jilin, China; 2 Henan University of Chinese Medicine, Zhengzhou, China

**Keywords:** hyper-inflammatory endotype, immune reprogramming, long COVID, pediatric pneumonia, secondary bacterial infection, two-hit model

## Abstract

Following the COVID-19 pandemic, the clinical patterns of pediatric respiratory infections have undergone significant changes, with increasing attention on the immunological imprint left by Post-Acute Sequelae of SARS-CoV-2 infection (PASC), commonly known as Long COVID. A perplexing clinical phenomenon has been observed: some children with a history of Long COVID exhibit a disproportionately severe inflammatory response and extensive lung injury when encountering common community-acquired pneumonia, such as that caused by *Mycoplasma pneumoniae* or *Streptococcus pneumoniae*, inconsistent with their pathogen load. This review aims to dissect this phenomenon and proposes the “Immune Priming and Two-Hit” model as its core pathophysiological framework. This model posits that the Long COVID state constitutes the “first hit,” establishing a “primed” or “hyper-reactive” immune baseline through viral persistence, trained immunity-induced monocyte reprogramming, and sustained endothelial dysfunction. Upon the “second hit” of a bacterial infection, this primed immune system triggers a dysregulated, synergistically amplified inflammatory cascade. The mechanisms involve the exponential release of cytokines such as Interleukin-6 (IL-6), IL-1β, and Tumor Necrosis Factor-*α* (TNF-α), inflammation-mediated immunothrombosis, and excessive activation of Neutrophil Extracellular Trap formation (NETosis), ultimately leading to severe outcomes like Acute Respiratory Distress Syndrome (ARDS) and necrotizing pneumonia. Consequently, identifying and defining this “Hyper-inflammatory endotype” is of critical clinical importance. We define it as an “endotype” to emphasize the distinct, host-determined pathophysiological mechanisms underlying it, rather than merely a collection of clinical manifestations. By monitoring biomarkers such as ferritin, D-dimer, lactate dehydrogenase (LDH), and lymphocyte counts, clinicians may be able to perform early risk stratification of these children. This approach not only facilitates a shift in therapeutic strategy from purely antimicrobial therapy to “host-directed therapy”—emphasizing the necessity of early, adequate corticosteroid use and consideration of anticoagulation—but also provides a new theoretical basis and intervention window for preventing long-term sequelae such as pulmonary fibrosis.

## 1 Introduction

The COVID-19 pandemic has fundamentally reshaped the global epidemiological landscape of respiratory infectious diseases, with profound implications for the pediatric population (1, 2). Although children typically exhibit milder symptoms during the acute phase of SARS-CoV-2 infection, its long-term consequences, known as Post-Acute Sequelae of SARS-CoV-2 infection (PASC) or Long COVID, have emerged as a significant public health concern (3, 4). These sequelae are diverse, ranging from persistent fatigue and cognitive dysfunction (colloquially termed “brain fog”) to long-term pulmonary impairment, suggesting that the viral infection may leave a lasting pathophysiological imprint in some children (3, 4). In the post-pandemic era, an alarming new trend has emerged in pediatric clinical practice across multiple regions: an unusual fluctuation in hospitalization and severity rates for pediatric community-acquired pneumonia (CAP), with a notable increase in infections associated with *Mycoplasma pneumoniae* and invasive Group A Streptococcus (2, 5). Against this backdrop, a clinical phenotype described as “refractory mixed pneumonia” has garnered widespread attention. It is characterized by a poor response to conventional antibiotic therapy, persistent high fever, and rapid progression of pulmonary consolidation on chest imaging, which can evolve into diffuse “white lung” within a short period. The severity of this condition far exceeds what can be explained by a single pathogen according to traditional understanding. This clinical dilemma prompts a central scientific question: is there an intrinsic link between this exceptionally severe pneumonia phenotype and a child’s prior history of COVID-19 infection?

Of course, other important macroscopic explanations for the fluctuating incidence of severe pediatric pneumonia in the post-pandemic era have been proposed. The “immunity debt” hypothesis suggests that prolonged public health interventions reduced children’s exposure to common pathogens, leading to an accumulation of susceptible individuals and subsequent infection peaks upon the resumption of social activities. Furthermore, the evolution of pathogens themselves, such as the enhanced virulence of specific Group A Streptococcus clones, is also considered a significant factor contributing to the rise in invasive infections. However, these explanations, based on population-level dynamics or pathogen characteristics, seem insufficient to fully address the core clinical puzzle this review focuses on: why, under similar epidemiological exposures, does a subset of children with a clear history of COVID-19 infection exhibit an abnormally intense inflammatory response pattern disproportionate to their pathogen load or conventional virulence? This phenomenon suggests that, beyond external factors, the host’s intrinsic immune baseline, shaped by prior infectious events, may play a more critical role in determining the ultimate severity of the disease. Therefore, the “Immune Priming and Two-Hit” model proposed in this review does not aim to negate the role of other macroscopic factors but rather seeks to focus on a specific, potentially overlooked, host-dependent mechanism to provide a more in-depth and specific pathophysiological framework for understanding this emerging severe clinical phenotype.

This review aims to systematically explore this issue by integrating evidence from post-viral immunology, bacterial infection pathology, and clinical medicine to first propose and elaborate on the “Immune Priming and Two-Hit” model. Our goal is to bridge the gap between the seemingly separate fields of post-viral sequelae and acute bacterial infections, providing a solid theoretical foundation for a deeper understanding of this emerging clinical challenge, establishing early diagnostic and stratification strategies based on biomarkers, and ultimately enabling a paradigm shift from traditional antimicrobial-centric models to precise, host-directed therapy.

### 1.1 Conceptual framework of the “two-hit” model

**Figure 1:**
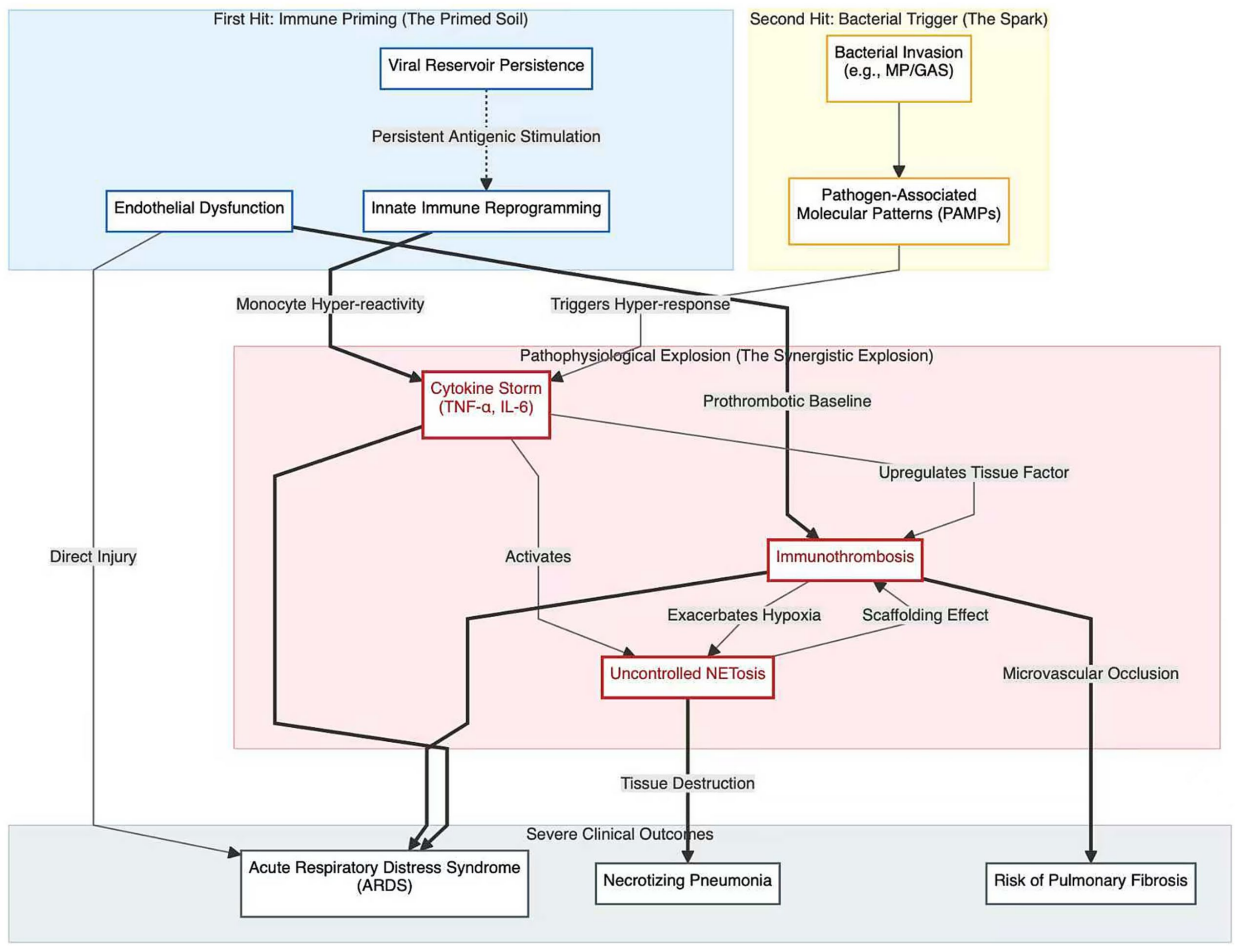
Conceptual framework of the “two-hit” model. This model illustrates that after experiencing long COVID (the “first hit”), a child’s immune system enters a “primed” state. This state is constituted by the persistence of viral reservoirs, “trained immunity” based on epigenetic reprogramming, and widespread endothelial dysfunction. Upon encountering a secondary bacterial infection (the “second hit”), the primed immune system triggers a dysregulated inflammatory explosion, with core mechanisms including a cytokine storm, immunothrombosis, and excessive NETosis, ultimately leading to severe clinical outcomes such as Acute Respiratory Distress Syndrome (ARDS) and necrotizing pneumonia.

In this framework, the “second hit” does not necessarily occur within the classic 1–3-week post-viral window; rather, it is conceptualized in the post-acute/Long COVID phase (weeks to months after SARS-CoV-2 infection). To visually articulate the core theory of this review, we have constructed a conceptual framework for the “two-hit” model. This framework clearly delineates the entire process from the immune-primed state of the “first hit” to the inflammatory explosion of the “second hit,” culminating in severe clinical consequences (Figure 1).

## 2 The hidden ember: the pathophysiological baseline of pediatric long COVID

The theoretical cornerstone of the “two-hit” model is the pathophysiological state created by the “first hit,” wherein PASC establishes a persistent, low-grade, yet functionally significant background of immune dysregulation in children. This state, akin to a hidden, continuously smoldering ember, may not manifest with severe clinical symptoms under normal circumstances but profoundly alters the body’s response pattern to subsequent infections. This section will delve into the three core pathophysiological mechanisms that constitute this “primed” state: the persistence of the virus or its components, epigenetic reprogramming of the innate immune system, and widespread endothelial dysfunction.

### 2.1 Viral persistence as a source of chronic antigenic stimulation

A growing body of evidence suggests that after the clearance of acute SARS-CoV-2 infection, viral nucleic acids or protein components (particularly the spike protein) may not be completely eliminated. Instead, they can form “viral reservoirs” in certain tissues, constituting a persistent source of chronic antigenic stimulation (6). These reservoirs are commonly found in sites with immune privilege or low clearance efficiency, such as gut-associated lymphoid tissue, the spleen, and lymph nodes. This continuous, low-level antigen exposure constantly stimulates Pattern Recognition Receptors (PRRs), leading to a state of low-grade, persistent activation of the immune system, especially innate immune cells. This state manifests as an elevated baseline level of inflammation, with various pro-inflammatory cytokines and chemokines maintained at higher levels than in healthy children, laying the foundation for subsequent excessive immune responses.

### 2.2 Trained immunity renders innate immune cells “hyper-reactive”

The chronic antigenic stimulation and low-grade inflammatory environment in the Long COVID state can induce an adaptive change in innate immune cells, particularly monocytes and macrophages, known as “trained immunity” (7). Trained immunity is a form of innate immune memory based on epigenetic reprogramming, which does not rely on antigen-specific lymphocytes. Specifically, histone modifications (e.g., enrichment of H3K4me3) and metabolic reprogramming (e.g., a shift from oxidative phosphorylation to glycolysis) cause lasting changes in the gene expression patterns of these cells, enabling them to mount a stronger and more rapid response to subsequent, unrelated pathogen stimuli (8). Importantly, mechanistic studies have provided direct evidence that coronavirus/SARS-CoV-2 infection can leave durable epigenetic “memory” signatures in innate immune cells and even their progenitors (e.g., hematopoietic stem and progenitor cells), consistent with a trained-immunity–like reprogramming that may persist for months after infection (9). Complementary work further supports airway-resident innate immune memory after SARS-CoV-2, in which alveolar macrophages retain a functionally relevant imprinted state that modulates inflammatory trajectories during subsequent heterologous respiratory challenges (10). This epigenetic-based innate immune memory is mechanistically similar to the remodeling seen in adaptive immunity; for instance, B cell immunophenotypes can also undergo lasting changes following certain viral exposures (11). In this state, monocytes are primed for a hyper-responsive state, with their inflammatory response threshold significantly lowered. When they re-encounter pathogen-associated molecular patterns (PAMPs) such as bacterial lipopolysaccharide (LPS) or lipoproteins, they trigger a burst-like production of pro-inflammatory cytokines (e.g., TNF-*α*, IL-6, IL-1β) far exceeding normal levels, thereby forming the cellular basis of the cytokine storm in the “second hit.” Interferon-*γ* (IFN-γ) has been identified as a key immunological marker in Long COVID. A recent study observed persistent, spontaneous IFN-γ production from peripheral blood mononuclear cells (PBMCs) in patients, even without pathogen stimulation. This response, mediated by CD8⁺T cells and antigen-presenting CD14⁺ cells, was associated with ongoing symptoms. IFN-*γ* levels declined with symptom improvement, indicating its potential as a biomarker for disease monitoring. While most studies focus on adults, pediatric data remain limited, and the role of IFN-γ in children with Long COVID is not yet fully elucidated (12).

### 2.3 Endothelial dysfunction establishes a systemic Prothrombotic environment

The SARS-CoV-2 virus, particularly its spike protein, can directly attack vascular endothelial cells expressing Angiotensin-Converting Enzyme 2 (ACE2), leading to widespread endothelial cell injury and dysfunction, a condition known as endotheliitis (6, 13). In patients with Long COVID, this endothelial injury can persist, as evidenced by sustained elevation of endothelial activation markers such as von Willebrand factor, soluble P-selectin, and VCAM-1 (13, 14). Damaged endothelial cells lose their original anticoagulant and anti-inflammatory properties, instead exposing tissue factor and downregulating thrombomodulin expression. This shifts the intravascular environment from a physiological anticoagulant state to a pathological prothrombotic state. This systemic prothrombotic background serves as a fertile ground for the formation of microclots, creating a critical vulnerability for the explosive development of immunothrombosis during the “second hit” (6).

## 3 The explosion: mechanisms of exacerbated bacterial pneumonia

If the Long COVID state is the “ember,” then a secondary bacterial infection is the “fuel,” and their encounter triggers a violent “explosion”—a disproportionately severe pulmonary injury. This process is not a simple superposition of pathogens but a multi-step, synergistically amplified pathophysiological cascade mediated by the host immune system. This chapter will focus on the three core driving mechanisms of this “explosion”: the synergistic amplification of the cytokine storm, catastrophic immunothrombosis, and the dysregulation and detrimental effects of NETosis.

### 3.1 Synergistic amplification of the cytokine storm as the engine of high fever and systemic inflammation

Against the “primed” immune background of Long COVID, when bacterial pathogens (the “second hit”) invade the lungs, their PAMPs, such as the lipoproteins of *Mycoplasma pneumoniae* or the peptidoglycan of *Streptococcus pneumoniae*, are rapidly recognized by “hyper-reactive” macrophages and monocytes. The resulting signal transduction is not a conventional, moderate inflammatory response but a “perfect storm” of cytokines. In animal models of viral-bacterial co-infection, it has been observed that a pre-existing viral infection significantly amplifies the host’s cytokine response to a subsequent bacterial challenge, leading to an exponential release of key pro-inflammatory cytokines such as Tumor Necrosis Factor-*α* (TNF-α), Interleukin-6 (IL-6), and Interleukin-1β (IL-1β) (7). This synergistic amplification effect directly leads to the clinically observed refractory high fever (body temperature persistently >39 °C) and the sharp, extreme elevation of inflammatory markers like C-reactive protein (CRP) and procalcitonin (PCT), constituting the core clinical features of “hyper-inflammatory endotype” pneumonia.

### 3.2 Immunothrombosis as a key driver of severe hypoxemia and pulmonary consolidation

Inflammation and coagulation are two tightly interwoven processes. In the “two-hit” scenario, this link is pathologically intensified, leading to what is termed immunothrombosis. On one hand, the persistent endothelial dysfunction and systemic prothrombotic state associated with Long COVID, as described in Section 2.3, provide a “fertile soil” for thrombosis (6). On the other hand, cytokines from the storm, such as TNF-*α* and IL-1β, can further activate endothelial cells and upregulate tissue factor expression, thereby potently initiating the extrinsic coagulation pathway (6, 13). Concurrently, activated platelets and neutrophils participate, collectively forming widespread microthrombi rich in fibrin and immune cells within the pulmonary microcirculation. These microthrombi occlude alveolar capillaries, severely disrupting the ventilation/perfusion (V/Q) match, and are the direct cause of the severe hypoxemia that is often refractory to conventional oxygen therapy. Radiologically, this extensive microvascular thrombosis, along with the ensuing alveolar exudate and edema, manifests as persistent ground-glass opacities and rapidly progressing, extensive pulmonary consolidation.

### 3.3 The double-edged sword of NETosis exacerbates tissue necrosis and structural lung damage

Neutrophil Extracellular Traps (NETs) are web-like structures composed of DNA, histones, and granular proteins released by neutrophils in response to pathogens, with the physiological function of trapping and killing them. However, in the pathological context of the “two-hit” model, the process of NETosis becomes completely dysregulated, turning into a “double-edged sword” that destroys tissue. The Long COVID state itself may be associated with neutrophil dysfunction and spontaneous NET formation. When a secondary bacterial invasion occurs, neutrophils, intensely activated by the cytokine storm, undergo massive, uncontrolled NETosis. In severe cases following other viral infections, such as dengue fever, excessive NETs have been closely linked to disease severity (15). Beyond associative observations, mechanistic and translational evidence implicates NETs as active drivers of acute lung injury/ARDS pathobiology, including endothelial/epithelial injury, capillary leak, and thromboinflammation, supporting their role in propagating severe respiratory failure (16). These overproduced NETs not only fail to effectively clear bacteria but their components, such as histones and proteases, are highly cytotoxic. They directly damage alveolar epithelial and endothelial cells, exacerbating lung injury and capillary leakage. Importantly for the “second hit,” experimental bacterial pneumonia studies further implicate NETs and extracellular histone H4 as exacerbating factors that intensify pneumonia-associated lung injury and necrosis, providing direct mechanistic support for how dysregulated NETosis could amplify tissue destruction during superimposed bacterial infection (17). More critically, the extensive NETs network can obstruct alveoli and small bronchioles, creating physical blockages and inducing thrombosis, ultimately promoting the development of Necrotizing Pneumonia. Clinically, this corresponds to the formation of pulmonary cavities and large pleural effusions on chest CT scans, marking the progression of the disease to a severe stage with irreversible destruction of lung architecture.

## 4 Clinical phenotype and diagnostic stratification

Translating the “two-hit” model from theory to clinical practice hinges on establishing a diagnostic stratification strategy that can effectively identify children with “hyper-inflammatory endotype” pneumonia. This requires not only re-examining the clinical presentation of traditional pneumonia but also integrating novel biomarkers to construct a multidimensional predictive model. The ultimate goal is to accurately triage patients at an early stage of the disease, providing a timely opportunity to initiate interventions targeting the host’s immune response.

### 4.1 Clinical portrait and proposed subtyping of “hyper-inflammatory endotype” pneumonia

Based on the “two-hit” model, we propose an initial classification of pediatric CAP into two main subtypes based on clinical and inflammatory features:

(1) Type A (conventional infection type): these patients have no clear history of COVID-19 infection or have resolved COVID-19 symptoms without progressing to Long COVID. Their bacterial or viral pneumonia typically exhibits a proportional inflammatory response to pathogen load and virulence, and they respond well to standard antimicrobial therapy.
(2) Type B (hyper-inflammatory/long COVID-associated type): these patients have a confirmed or suspected history of COVID-19 and ongoing Long COVID symptoms. In this context, a secondary bacterial infection may trigger an exaggerated inflammatory response disproportionate to pathogen burden, often leading to refractory symptoms and rapid progression.

### 4.2 Clinical prediction models and biomarkers for risk stratification

To implement the aforementioned subtyping, a practical clinical prediction model is needed. Existing research has demonstrated that machine learning models integrating multi-source data (including clinical indicators, laboratory tests, and imaging features) can effectively stratify the risk of severe pneumonia in children (18). We propose to build upon this by further integrating biomarkers that reflect the core mechanisms of the “two-hit” model to enhance the model’s specificity and accuracy.

(1) Reinterpretation of conventional markers: in the context of the “hyper-inflammatory endotype,” the extreme elevation of some conventional inflammatory markers has special diagnostic value. In particular, a sharp rise in serum ferritin [e.g., ≥500 ng/mL (19)] is not only a strong inflammatory marker but also a key warning signal for Macrophage Activation Syndrome (MAS) or a cytokine storm (6).
(2) Novel markers reflecting tissue damage and coagulation activation: a significant increase in Lactate Dehydrogenase (LDH) reflects widespread cellular damage and tissue necrosis, especially in the lungs, making it a reliable indicator of the severity of lung injury (7). Concurrently, a progressive increase in D-dimer levels directly points to the activation of immunothrombosis and is a key factor in predicting thrombotic complications and disease severity (18). To improve cross-laboratory interpretability, D-dimer should preferably be expressed relative to the assay-specific upper limit of normal (ULN) and interpreted dynamically (trajectory), rather than implying a universal absolute cutoff (20).
(3) Indicators for assessing immune status: persistent lymphopenia, especially after excluding the transient decrease caused by the viral infection itself, often suggests that the host’s immune system is in a state of exhaustion and has been associated with worse clinical outcomes, supporting its role as a supportive indicator of impaired immune status rather than an endotype-defining criterion on its own (21).
(4) Dynamic imaging assessment: lung ultrasound (LUS), as a non-invasive, real-time, point-of-care tool, is superior to traditional chest X-rays for dynamically monitoring pulmonary lesions (such as the extent of consolidation and the volume of pleural effusion). It can provide more timely information for assessing disease progression and treatment response (22).

By integrating a history of COVID-19 infection with the aforementioned biomarkers and imaging data, it is feasible to construct a clinical decision support system capable of early and accurate identification of children with “hyper-inflammatory endotype” pneumonia.

However, it must be recognized that the currently proposed combination of biomarkers (ferritin, LDH, D-dimer, etc.) serves more as general indicators of a “hyper-inflammatory state” rather than specific markers defining the “hyper-inflammatory endotype.” To fundamentally establish its status as an “endotype,” future research must delve into the mechanistic level to develop and validate specific biomarkers that directly reflect the core pathophysiological links of the “two-hit” model. This involves two main directions: (1) Biomarkers for assessing the “trained immunity” state: For example, by establishing standardized *in vitro* functional assays, peripheral blood mononuclear cells (PBMCs) from patients can be isolated and re-stimulated (e.g., with bacterial LPS), followed by quantification of the pro-inflammatory cytokines (e.g., TNF-*α*, IL-6) they produce. A “primed” monocyte will exhibit a significantly stronger response than a normal control, serving as direct functional evidence of a “hyper-reactive” state. (2) Specific biomarkers reflecting NETosis activation: While levels of cell-free DNA or histones in plasma may be elevated, they lack specificity. More precise indicators should include the detection of specific degradation products of NETs, such as myeloperoxidase-DNA (MPO-DNA) complexes or citrullinated histone H3 (H3Cit). The development and validation of these mechanistic biomarkers are a critical step in transforming the “hyper-inflammatory endotype” from a clinical observational concept into a precisely targetable pathophysiological entity.

### 4.3 Differential diagnosis: from phenomenological comparison to mechanistic tracing

The differential diagnosis of “hyper-inflammatory endotype” pneumonia is extremely challenging, as its clinical presentation (high fever, high inflammatory markers, multi-organ dysfunction) overlaps significantly with various pediatric critical illnesses. However, the core of the differentiation should not merely rest on comparing clinical phenomena but must delve into the initiating pathophysiological steps, i.e., tracing back to its unique precondition of “post-viral immune priming”.

(1) Mechanistic distinction from hemophagocytic lymphohistiocytosis (HLH): although both share the final common pathway of a cytokine storm, their “ignition” mechanisms are fundamentally different. Primary HLH originates from genetic defects in immune regulatory genes, such as the perforin pathway, while secondary HLH is often associated with Epstein–Barr virus (EBV) infection or rheumatic diseases. In contrast, the “defect” in “hyper-inflammatory endotype” pneumonia does not stem from classic immune regulatory gene mutations but is an acquired “functional reprogramming” (e.g., trained immunity) imprinted by a specific virus (SARS-CoV-2), which causes an abnormal response to common bacterial infections. Therefore, a child lacking HLH-associated gene mutations or evidence of active EBV infection, but with a clear history of Long COVID symptoms, is more likely to be diagnosed with the “hyper-inflammatory endotype”.
(2) Mechanistic distinction from kawasaki disease (KD): the hyper-inflammatory state of “hyper-inflammatory endotype” pneumonia is similar to that of KD, especially the shock-associated form. However, the essence of KD is considered to be an acute systemic vasculitis, primarily affecting small and medium-sized arteries, triggered by an unknown pathogen. The vascular injury (endotheliitis) in the “hyper-inflammatory endotype” has a different temporal trajectory: it is a chronic, persistent endothelial dysfunction formed during the “first hit” (Long COVID), which is then dramatically amplified by the inflammatory storm during the “second hit” (bacterial infection), leading to immunothrombosis. Therefore, a child lacking the typical clinical criteria for KD (e.g., conjunctival injection, coronary artery dilation) but presenting with progressively rising D-dimer levels and severe hypoxemia has a pathological basis more consistent with the “two-hit” model.
(3) Mechanistic distinction from toxic shock syndrome (TSS): the core pathophysiology of TSS is the action of bacterial exotoxins as “superantigens,” which bypass conventional antigen presentation pathways to directly and non-specifically activate a large number of T lymphocytes, representing a catastrophic activation of the adaptive immune system. In contrast, the core driving force of the “hyper-inflammatory endotype” originates from the excessive response of the innate immune system (especially monocyte-macrophages) to conventional bacterial PAMPs. In short, TSS is a “toxin-driven adaptive immunity catastrophe,” whereas the “hyper-inflammatory endotype” is “innate immune dysregulation in a primed state”.

In summary, the differential diagnosis of “hyper-inflammatory endotype” pneumonia requires a shift in clinical thinking from a “downstream” comparison of clinical phenomena to an “upstream” tracing of etiology and initiating mechanisms. This elevates the importance of a detailed inquiry into the child’s history of COVID-19 infection and Long COVID-related symptoms (e.g., persistent fatigue, cognitive impairment) to an unprecedented level, making its value equivalent to tracing family history in diagnosing HLH or searching for an infection source in diagnosing TSS.

### 4.4 Model limitations and current controversies

It must be acknowledged that the “Immune Priming and Two-Hit” model, as a host-centric theoretical framework, has explanatory boundaries. It aims to clarify how a specific host background can amplify the severity of an infection, but it cannot be entirely independent of the biological properties of the pathogen itself. The increase in severe pediatric infections in the post-pandemic era is a complex public health phenomenon, for which there are several other equally important explanations in the academic community.

A core point of contention is that some researchers believe the recent global surge in invasive Group A Streptococcus (iGAS) infections may be primarily driven by the evolution of the pathogen itself, rather than a general change in host immune status. For example, evidence suggests that the global spread of a newly emerged M1UK clone carrying specific virulence genes may be associated with an increase in the severity of iGAS disease (5). These hypervirulent strains may have acquired enhanced immune evasion or toxin-producing capabilities, enabling them to cause severe disease even in hosts without “immune priming.” Similarly, for *Mycoplasma pneumoniae*, the evolution of its drug resistance and changes in circulating strains could also affect its pathogenicity.

Therefore, attributing the severe pneumonia of the post-pandemic era entirely to host factors caused by Long COVID would be a one-sided view. A more comprehensive perspective should be that disease severity is the final outcome of the interaction between host susceptibility (the “soil”) and pathogen virulence (the “seed”). The “two-hit” model proposed in this review focuses on explaining why the “soil” has become unusually “fertile,” while the “pathogen-centric” view emphasizes the malignancy of the “seed” itself. Future research must integrate multi-dimensional data, including host immune profiling and pathogen genomic sequencing, to fully depict this complex picture and ultimately clarify the respective roles played by the host and the pathogen in the tragedy of each severe case.

## 5 Therapeutic and management implications

Based on the deep understanding of the pathophysiological mechanisms provided by the “two-hit” model, we must fundamentally rethink the treatment strategies for “hyper-inflammatory endotype” pneumonia. The traditional treatment paradigm has proven inadequate in addressing this new clinical challenge. This chapter aims to propose a more targeted, three-dimensional therapeutic framework based on the new mechanisms, with its core being a shift from solely “killing the pathogen” to “modulating the host,” thereby providing a new clinical pathway to improve patient outcomes.

### 5.1 A fundamental shift in therapeutic philosophy: from “antimicrobial-centric” to “host-directed”

For “hyper-inflammatory endotype” pneumonia, the core driver of clinical deterioration is not the bacteria itself, but the host’s dysregulated immune response. Therefore, clinicians must recognize that when a child’s condition does not improve or even worsens after receiving standard, adequate antibiotic therapy, the primary consideration may not be bacterial resistance or the need to escalate antibiotics (23, 24). In such cases, simply switching or adding broader-spectrum antibiotics is often futile and may even exacerbate immune dysregulation by disrupting the gut microbiota. The correct therapeutic approach should immediately shift to “Host-Directed Therapy” (HDT), meaning the focus of treatment should rapidly move from “antimicrobial” to “anti-inflammatory,” “anticoagulant,” and “immunomodulatory” strategies. This conceptual shift is the cognitive prerequisite for implementing all subsequent effective therapeutic measures.

Importantly, proposing a host-primed “two-hit” phenotype does not diminish the primacy of pathogen-directed management. Severe pediatric pneumonia may be driven predominantly by a host-amplified inflammatory–endothelial response on a primed baseline, or by pathogen factors such as hypervirulence and toxin-mediated injury, and these drivers can coexist (25–27). Therefore, timely microbiological evaluation and prompt effective antimicrobials remain foundational in all cases, and source control and anti-toxin strategies should be prioritized when clinically indicated (1, 2). In this context, HDT should be framed strictly as an adjunct; its timing and priority should be individualized according to whether the observed hyperinflammation/immunothrombosis appears disproportionate to the inferred pathogen burden and clinical trajectory (25). Even in pathogen-driven one-hit cases, host modulation may remain relevant as an adjunct for complications (e.g., shock/ARDS), but must not delay pathogen-directed escalation.

### 5.2 Immunomodulatory therapy: an exploratory strategy based on pathophysiological inference

Based on the “two-hit” model, where the core driver of the disease is the host’s dysregulated immune response, immunomodulatory therapy naturally becomes a central strategy to halt its progression. Among these, corticosteroids, as broad-spectrum and potent anti-inflammatory drugs, represent a theoretically attractive intervention option for early application. Based on the pathophysiology of the cytokine storm and rapid progression of pulmonary consolidation, the use of systemic corticosteroids (e.g., methylprednisolone) at a very early stage (e.g., within 3–5 days of onset) after identifying high-risk children—before irreversible structural changes such as necrosis or organization appear on lung imaging—could be a potential strategy to block the inflammatory cascade and protect lung tissue. In studies of some invasive bacterial infections, it has also been observed that adjunctive therapies, including corticosteroids, are widely used in cases with severe systemic reactions (5).

However, it must be emphasized that this strategy, based on pathophysiological inference, is a double-edged sword in clinical practice and is not without significant risks. When hypervirulent or toxin-mediated infection is suspected (e.g., bacteremia, necrotizing pneumonia, or toxic shock features), immunomodulation should not delay escalation of pathogen-directed therapy and should be restricted to guideline-consistent management of complications with careful individualized risk–benefit assessment (25–27). In the context of an active bacterial infection, the use of corticosteroids may suppress the host’s effective pathogen clearance mechanisms, leading to bacterial dissemination or reactivation of latent viruses (such as cytomegalovirus). Furthermore, potent immunosuppression may increase the risk of secondary fungal infections and could mask the clinical signs of worsening infection, complicating patient assessment.

Therefore, all current recommendations for the use of immunomodulatory therapy in “hyper-inflammatory endotype” pneumonia must be considered exploratory. The efficacy and safety of these strategies lack direct evidence from randomized controlled trials (RCTs) specifically targeting this population. Clinicians must conduct an extremely cautious, individualized benefit–risk assessment when considering their use. Future research urgently needs to clarify the optimal timing, dosage, and duration of immunomodulatory therapies [including corticosteroids, IVIG, or biologics like tocilizumab (28)] and their true clinical value through well-designed clinical trials.

### 5.3 Cautious evaluation of anticoagulation: balancing microcirculatory protection and bleeding risk

Given the central role of immunothrombosis in this pathological process and the inherent prothrombotic state of Long COVID (6), the potential role of anticoagulant therapy and its benefit–risk balance have become a complex issue requiring urgent evaluation. Theoretically, for children with significantly and persistently elevated D-dimer levels, early initiation of prophylactic-dose anticoagulation (e.g., low-molecular-weight heparin) might improve pulmonary perfusion, alleviate severe hypoxemia, and potentially create a more favorable microenvironment for the repair of damaged lung tissue by inhibiting widespread microthrombus formation in the pulmonary microcirculation; however, “markedly elevated” D-dimer should be interpreted relative to the assay-specific upper limit of normal (ULN) and in conjunction with clinical evidence of thrombotic risk, and anticoagulation should not be triggered by D-dimer alone.

However, this theoretical benefit must be weighed against a major, and potentially fatal, risk: bleeding. In “hyper-inflammatory endotype” pneumonia, the intense inflammatory response and NETosis can lead to tissue necrosis and cavity formation in the lungs. In such circumstances, the use of anticoagulants is highly likely to induce uncontrollable intrapulmonary hemorrhage, leading to catastrophic consequences.

Therefore, anticoagulant therapy should by no means be considered a routine recommendation. The decision must be individualized and requires a high degree of clinical vigilance. Clinicians must dynamically assess D-dimer levels, platelet counts, and changes on chest CT scans under close monitoring. Anticoagulation should only be cautiously considered within a very short “therapeutic window” in a very small subset of specific patients where the risk of thrombosis is believed to far outweigh the risk of bleeding, and where lung imaging does not suggest a clear tendency for cavitation or necrosis. Similarly, the definitive role and benefit–risk ratio of this strategy must ultimately be answered by evidence from future RCTs.

In presentations that appear predominantly pathogen-driven, anticoagulation—if considered—should follow established pediatric thrombosis indications and bleeding-risk stratification, and must never substitute for or distract from rapid targeted antimicrobials and source control (25, 29).

### 5.4 Proposed clinical pathway: a decision-making process for stratified management

**Figure 2:**
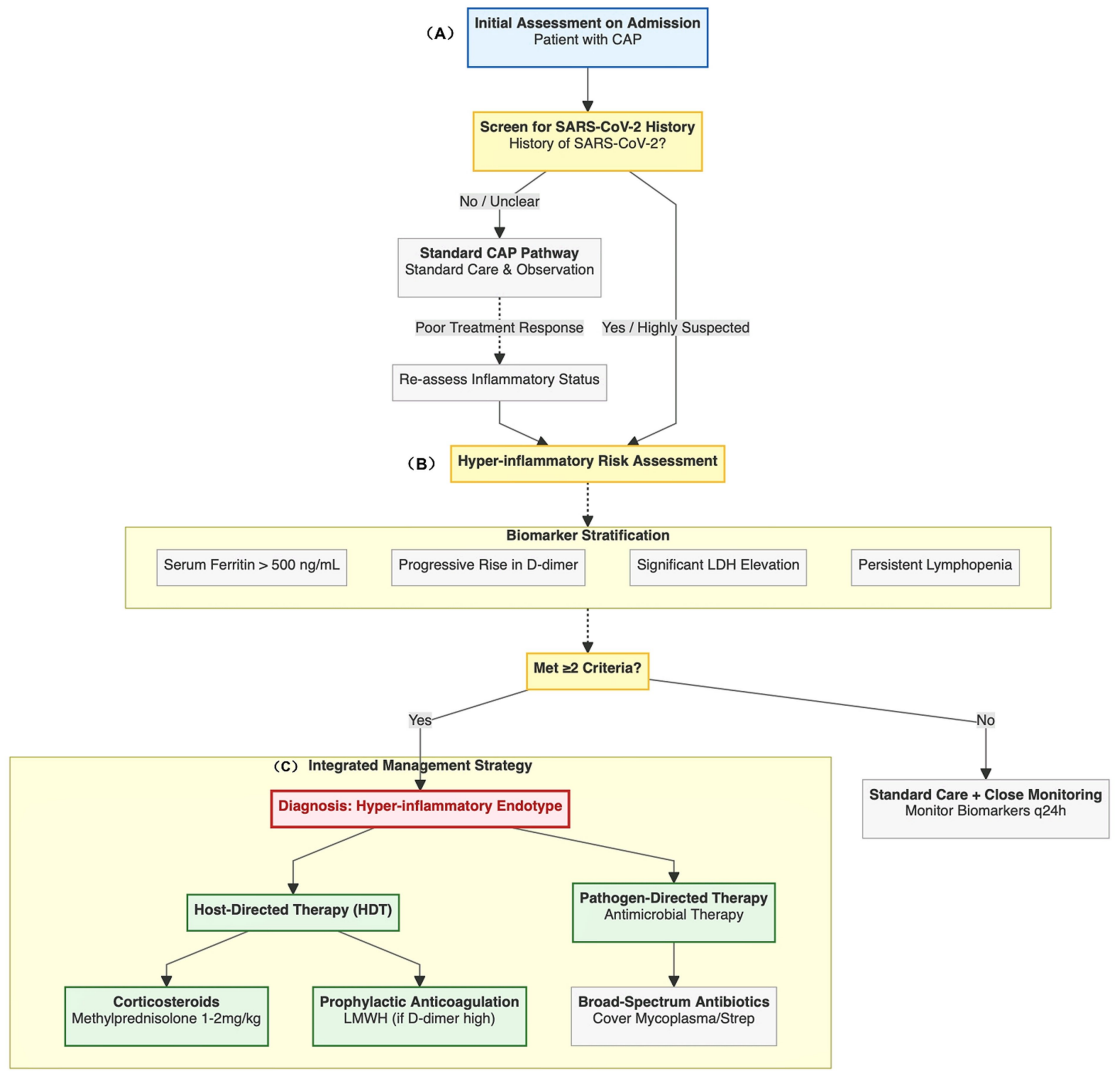
Proposed clinical decision-making pathway for the identification and management of the hyper-inflammatory endotype in pediatric pneumonia. **(A)** This flowchart outlines a stratified diagnostic and therapeutic approach. The process begins with screening all children admitted for community-acquired pneumonia (CAP) for a history of SARS-CoV-2 infection. **(B)** Children with a prior history of infection or those who do not respond to standard therapy undergo a hyper-inflammatory risk assessment based on key biomarkers (ferritin, D-dimer, LDH, and lymphocyte count). **(C)** Meeting ≥2 criteria should be interpreted as a working risk-enrichment flag (rather than a validated diagnostic rule) of the “hyper-inflammatory endotype,” indicating a shift in treatment strategy from standard care to an integrated management approach. This includes host-directed therapy (HDT)—characterized by early corticosteroids and conditional anticoagulation—alongside broad-spectrum antimicrobial coverage, while recognizing that the specific cutoffs and decision rules require prospective calibration and validation.

To translate the concept of “host-directed therapy” into an actionable clinical tool, we have constructed a proposed decision-making pathway for stratified management. This pathway is designed to help clinicians systematically assess the risk of the “hyper-inflammatory endotype” in children with suspected severe pneumonia and initiate corresponding stratified treatment strategies (Figure 2).

## 6 Long-term prognosis and follow-up

For children who survive the “two-hit” storm, hospital discharge does not signify the end of their recovery but merely the beginning of a long-term rehabilitation process. The severe inflammation and tissue damage can leave profound long-term sequelae that may continue to affect the child’s health for months or even years. Therefore, establishing a systematic, long-term follow-up plan is crucial, focusing on the monitoring and intervention of two major long-term complications: pulmonary fibrosis and persistent airway hyper-responsiveness.

### 6.1 Pulmonary fibrosis as a significant risk after a hyper-inflammatory storm

The repair mechanism following severe pulmonary inflammation, especially when accompanied by Acute Respiratory Distress Syndrome (ARDS) and necrotizing pneumonia, is often aberrant. Normal alveolar structural repair is replaced by excessive fibroblast proliferation and massive deposition of extracellular matrix (e.g., collagen), ultimately leading to pulmonary fibrosis. Research in diseases such as idiopathic pulmonary fibrosis (IPF) has clearly established a close crosstalk between inflammatory and pro-fibrotic signaling pathways (24). Therefore, the risk of pulmonary fibrosis must be considered in these children. Regular, long-term follow-up is recommended after discharge, which should include at least: (1) Pulmonary Function Tests: focusing on indicators such as Vital Capacity (VC), Forced Vital Capacity (FVC), and Forced Expiratory Volume in 1 s (FEV1) to assess the degree of restrictive ventilation dysfunction. (2) High-Resolution CT (HRCT): to visually assess fibrotic changes in the lung interstitium, such as reticular patterns or honeycombing. These examinations should be performed at 3 months, 6 months, 1 year post-discharge, and periodically thereafter as clinically indicated.

### 6.2 Persistent airway hyper-responsiveness as the root of chronic cough

Although post-infectious cough and transient airway hyper-responsiveness can occur after severe pneumonia in general, we propose that the COVID-19 “first hit” may increase both susceptibility and persistence of this phenotype in a subset of children (30, 31). COVID-19–associated cough has been linked to a cough-hypersensitivity framework involving neuro–immune dysregulation together with ongoing airway inflammation and epithelial dysfunction, which may lower the threshold for sensory nerve hyperexcitability (30). Within our two-hit model, the “second hit” of severe bacterial pneumonia superimposed on this primed baseline may therefore prolong airway remodeling and cough hypersensitivity, resulting in more persistent AHR and chronic cough than would be expected from bacterial infection alone (30–32). Many children who have experienced severe pneumonia exhibit a long-term, recurrent cough after discharge, especially during exercise, exposure to cold air, or upon subsequent minor respiratory infections. This phenomenon is typically attributed to post-infectious Airway Hyper-responsiveness (AHR). In the context of “hyper-inflammatory endotype” pneumonia, this AHR may be more pronounced and persistent. The intense inflammatory response not only damages the alveoli but also affects the airway epithelium, leading to impaired epithelial barrier function, exposure of sensory nerve endings, and airway smooth muscle remodeling. These pathological changes collectively lead to an abnormally increased reactivity of the airways to various stimuli. Furthermore, Long COVID itself can be associated with long-term cough symptoms (3, 4), and the superposition of these two conditions makes the symptoms more complex. Therefore, the chronic cough in these children should not be simply attributed to a “weak constitution” or an “unresolved cold.” The possibility of AHR should be considered, and appropriate evaluation and intervention should be undertaken, such as using a bronchial challenge test for a definitive diagnosis and considering treatments like inhaled corticosteroids to control airway inflammation. Accordingly, the evaluation and symptomatic management steps described above reflect general best practice for post-severe-pneumonia cough/AHR, but they are particularly important to implement early in children with compatible post-COVID history, in whom cough hypersensitivity and airway remodeling may be amplified or prolonged by the primed “first-hit” baseline (30, 31).

## 7 Conclusion and future perspectives based on the “two-hit” model

The core of this review has been to construct and systematically elaborate on the “Immune Priming and Two-Hit” model, aiming to provide an integrated pathophysiological explanation for the new phenotype of severe pediatric pneumonia observed in the post-pandemic era. We have argued that Long COVID (PASC) is essentially a profound immune remodeling process that sets a “high-risk” immune baseline for the body through viral persistence, trained immunity, and endothelial dysfunction. When a secondary bacterial infection is encountered, this primed system triggers an inflammatory explosion centered on a cytokine storm, immunothrombosis, and dysregulated NETosis. Based on this core model, we call for a paradigm shift in clinical practice and propose the following three key future research directions.

### 7.1 Clinical translation of the model: from risk stratification to preventive strategies

The primary task for the future is to translate this model into life-saving clinical strategies. We not only need to validate the predictive value of a history of COVID-19 infection and related biomarkers (ferritin, D-dimer, LDH) for pneumonia severity through large-scale prospective cohort studies, but also to define risk-stratified preventive strategies that can be implemented safely during structured follow-up after the “first hit” (e.g., prior to respiratory infection seasons). Importantly, this does not imply routine prophylactic antibiotics for children with Long COVID; antibiotics should remain reserved for confirmed or strongly suspected bacterial infection, consistent with antimicrobial stewardship principles (33). Time-after-exposure versus age-related immune maturation. A key unresolved issue for clinical translation is whether the proposed hyper-inflammatory endotype is predominantly driven by time since SARS-CoV-2 infection (a post-viral immune “imprint” that may attenuate over months) or by age-related immune maturation, which can shift inflammatory thresholds and potentially reduce the clinical expression of this phenotype as children grow. Current pediatric evidence remains insufficient to disentangle these effects, largely because most studies lack repeat immunophenotyping stratified simultaneously by chronological age and time since infection. Nevertheless, longitudinal pediatric cohorts have reported heterogeneous recovery trajectories, with a subset of children meeting Long COVID research definitions up to 24 months after infection, consistent with the possibility that immune dysregulation may persist in a proportion of individuals (34). Mechanistically, durable innate immune reprogramming (“trained immunity”) and epigenetic remodeling of myeloid compartments have been described after COVID-19, with immune alterations persisting for months to approximately one year, supporting the biological plausibility of longer-lived endotypes (10, 35). In parallel, the pediatric immune system undergoes marked postnatal maturation with age-dependent differences in innate and adaptive responsiveness, providing a second, non-mutually exclusive explanation for age-associated shifts in inflammatory phenotypes (36, 37). Therefore, future prospective cohorts should adopt a factorial design (age×time-since-infection) with repeated immune profiling and standardized follow-up for severe bacterial pneumonia outcomes, to define the optimal screening window, biomarker cutoffs, and timing of risk-stratified monitoring and preventive strategies for children at highest risk. This raises a critical scientific question: can we mitigate downstream risk for the subgroup of children with persistent Long COVID symptoms who appear immunologically “primed”? At this stage, we do not recommend prophylactic pharmacologic interventions in routine care. Instead, future research could evaluate—under rigorously designed, safety-focused clinical trials—whether targeted host-modulating approaches can normalize dysregulated immune signatures and reduce the probability or severity of a subsequent “second hit.” In parallel, near-term preventive efforts should prioritize non-pharmacologic and low-risk measures (e.g., vaccination optimization, management of comorbid airway disease, and structured early-warning pathways during infection seasons), while any immune-modulating or microbiome-directed interventions should be treated as hypothesis-generating and trial-dependent rather than prescriptive clinical recommendations.

### 7.2 Deepening the mechanistic understanding of the model: seeking more precise therapeutic targets

Although this model delineates the core pathophysiological axis, its internal molecular details remain to be elucidated. Future basic research should utilize single-cell multi-omics technologies to precisely map the dynamic evolution of the immune microenvironment in the lungs during the “second hit.” Dissecting the specific roles of different immune cell subsets (e.g., different phenotypes of macrophages and neutrophils) in amplifying inflammation will provide key clues for developing targeted therapeutic drugs that are more precise and have fewer side effects than non-specific cytokine inhibitors. Furthermore, for children with the “hyper-inflammatory endotype,” there is an urgent need for well-designed RCTs to determine the optimal timing, dosage, and duration of immunomodulatory therapies (especially corticosteroids) and to evaluate the precise role and benefit–risk ratio of anticoagulant therapy.

### 7.3 Theoretical expansion of the model: a general paradigm for post-viral sequelae?

The most thought-provoking potential of the “Immune Priming and Two-Hit” model lies in its universality. SARS-CoV-2 may not be the only virus capable of leaving a lasting “immune imprint.” Could many other viral infections that cause persistent symptoms, such as Epstein–Barr virus (EBV), influenza virus, and even Respiratory Syncytial Virus (RSV), also establish a dysfunctional immune baseline in some individuals through similar mechanisms (e.g., viral latency, trained immunity)? Is the currently observed global increase in pediatric invasive Group A Streptococcus infections related not only to COVID-19 but also reflective of a more susceptible host immune background shaped by the co-circulation of multiple viruses? Expanding this model from being “COVID-specific” to a “general paradigm of post-viral immune remodeling” would provide a novel theoretical framework for understanding the complexity of various viral-bacterial co-infections, which is undoubtedly an exciting research direction in the field of infection immunology. Only through the close integration of clinical and basic research can we ultimately turn this emerging challenge into an opportunity to improve child health.
